# PCNP is a novel regulator of proliferation, migration, and invasion in human thyroid cancer

**DOI:** 10.7150/ijbs.70394

**Published:** 2022-05-16

**Authors:** Ya-Ge Chen, Hong-Xia Liu, Ya Hong, Peng-Zhen Dong, Shi-Yu Liu, Ying-Ran Gao, Dan Lu, Tao Li, Da-Yong Wang, Dong-Dong Wu, Xin-Ying Ji

**Affiliations:** 1 Henan International Joint Laboratory for Nuclear Protein Regulation, School of Basic Medical Sciences, Henan University, Kaifeng, Henan 475004, China; 2 School of Stomatology, Henan University, Kaifeng, Henan 475004, China; 3 Kaifeng Municipal Key Laboratory of Cell Signal Transduction, Henan Provincial Engineering Centre for Tumor Molecular Medicine, Henan University, Kaifeng, Henan 475004, China; 4 Kaifeng Key Laboratory of Infection and Biological Safety, School of Basic Medical Sciences, Henan University, Kaifeng, Henan 475004, China; 5 The First Affiliated Hospital of Henan University, Kaifeng, Henan 475001, China

**Keywords:** PCNP, thyroid cancer, cell cycle, apoptosis, autophagy

## Abstract

Thyroid cancer (TC) has increased globally, with a prominent increase in small, papillary thyroid cancers. PEST-containing nuclear protein (PCNP), a nuclear protein, has been found to be associated with human cancers in recent years. However, the role and molecular mechanism of PCNP in thyroid cancer remain underexplored. In the present study, the results showed that the expression levels of PCNP in human thyroid tissues were higher than those in adjacent non‐tumor tissues. Overexpression of PCNP reduced the proliferation, migration, and invasion of human thyroid cancer cells and down‐regulation of PCNP showed reverse effects. In addition, PCNP regulated cell cycle arrest through modifications in the expression of cell cycle regulating genes and PCNP affected apoptosis via activation of ERK/JNK/p38 pathway in thyroid cancer cells. Moreover, PCNP overexpression promoted autophagy by reducing the expression levels of Wnt/β‐catenin pathway in TC cells, however, PCNP knockdown had opposite effects. Furthermore, PCNP overexpression reduced the growth of xenografted human thyroid cancer, whereas PCNP knockdown showed opposite trends. In conclusion, *in vitro* and *in vivo* data demonstrate that PCNP as a tumor suppressor gene may serve as a novel prognostic and potential therapeutic marker in human thyroid cancer.

## Introduction

Thyroid cancer (TC) is the most common type of endocrine malignancy, and its incidence has rapidly increased in recent decades^1^. According to the cancer statistics, the incidence rate of thyroid cancer is ranked ninth with the global incidence ratio of women is 10.2 per 100,000 people, which is three times that of men, and the incidence of thyroid cancer has continued to increase in many countries since the 1980s^2^-^4^. Metastatic thyroid carcinoma remains a serious clinical issue with a poor prognosis despite intensive multimodal therapy of surgical resection, radiotherapy and levothyroxine treatment^5^. However, the underlying molecular mechanism responsible for the development of TC remains obscure. Therefore, identification of new molecular players involved in carcinogenesis and understanding their factual biological functions might be a key factor for effective selection of groups of risk among patients and administration of more personalized treatments.

PEST-containing nuclear protein (PCNP), characterized by a PEST motif rich in proline (P), glutamic acid (E), serine (S), and threonine (T), is firstly identified by database mining^6^. The PCNP protein with the PEST motif is highly subjected to ubiquitination^7^. As one of a well-established post-translational modification, ubiquitination is responsible for the protein substrate levels as well as the localization and activity of key protein kinases in many signaling pathways^8^. As such, PCNP may possess the ability to act as a transcriptional factor, cell cycle regulatory protein and tumor regulating nuclear protein. Actually, recent studies have indicated that PCNP may be involved in some aspects of tumorigenesis. For example, PCNP was demonstrated as a tumor suppressor in neuroblastoma cancer^9^ and a tumor promoter in ovarian cancer^10^ and lung adenocarcinoma cancer^11^. However, the role of PCNP in thyroid carcinoma still remains poorly understood.

In this study, PCNP was characterized to be highly expressed in thyroid tumors and cell lines. Overexpressed PCNP repressed the proliferation, migration, and invasion as well as cell cycle but promoted apoptosis and autophagy in thyroid carcinoma. *In vivo* and *in vitro* experiments deepen our understanding of the pivotal roles of PCNP in thyroid carcinoma progression and also provide new ideas for anti-cancer intervention.

## Materials and Methods

### Cell lines and cell culture

Human thyroid carcinoma cell lines (TC, ARO, TPC-1, FTC-133) and thyroid epithelial cell line Nthy-ori3-1 were obtained from Cobioer Biosciences (Nanjing, Jiangsu, China). Cells were maintained in RPMI-1640 medium (Gibco, Hangzhou, China) supplemented with 10% fetal bovine serum (Gibco Invitrogen, Grand Island, NY, United States), 100 μg/ml streptomycin, and 100 U/ml penicillin. All cell lines were authenticated by Short Tandem Repeat (STR) assay and cultured at 37 °C in a humidified atmosphere of 5% CO_2_.

### Patients and tissues samples

Human thyroid cancer tissue microarray was provided by National Human Genetic Resources Sharing Service Platform (Shanghai, China). The expression levels of PCNP were detected by immunohistochemical (IHC) on the microarray containing 91 human thyroid cancer specimens and corresponding non-tumor thyroid tissues (These clinical data were listed in Table 1). Tissue specimens used in this study were obtained from the First Affiliated Hospital of Henan University and the informed consents were obtained from all patients. Four fresh specimens collected at the time of surgery were used to detect the protein levels of PCNP. Pathological classification and clinical staging were based on the American Joint Committee on Cancer (AJCC) criteria.

### Overexpression and knockdown of PCNP

The PCNP overexpression plasmid, knockdown plasmid (sh-PCNP) and their respective negative control (NC) counterparts were purchased from GeneChem (Shanghai, China). To construct stable cell lines, the transfected cells were screened, respectively, by 800 μg/mL G418 (Solarbio, Shanghai, China) and 5 µg/ml puromycin (Solarbio, Shanghai, China). The un-transfected tumor cells were used as controls. The localization of PCNP in cancer cells was observed under a fluorescent microscope (Eclipse Ti, Nikon, Melville, NY, USA). The efficiency of PCNP-overexpression or -knockdown was monitored by Western blot assay.

### RNA extraction and quantitative RT-qPCR assay

TRIzol reagent was used to extract total RNA for RT-qPCR assay. Total RNA (1 μg) was reversely transcribed into cDNA using the primeScript RT reagent kit with gDNA Eraser (Takara Biotechnology, Co., Ltd., Dalian, China). The RT-PCR reaction was performed as previously described. For the PCNP gene, the forward primer was ATAGGATCCAAAATGGCGGACGGGAAGGCG and the reverse primer was CCGAAGCTTTTAATTGTCTTGGTCATGGAC. For the glyceraldehyde-3- phosphate dehydrogenase (GAPDH) gene, the forward primer was TATGACAACGAATTTGGCTACAG and the reverse primer was GATGGTACATGACAAGGTGC. The PCR amplification procedure was performed in 20 µL reaction reagents by 40 cycles with pre-denaturation at 95 °C for 10 min, denaturation at 60 °C for 60 s, and annealing and extension at 72 °C for 1 min.

### Cell viability and proliferation assays

Cell viability was assessed using MTS assay kit (Promega). Briefly, cells from different groups were seeded in 96‐well plates at 2 × 10^3^ cells/well. After incubation, the MTS reagent was added and incubated for 4 h at 37 °C. Absorbance was determined at 490 nm using SpectraMax M2 plate reader. The cell proliferation was tested by 5-ethynyl-2′-deoxyuridine (EdU) assay using Cell-Light EdU DNA Cell Proliferation Kit (RiboBio, Shanghai, China) according to the manufacturer's instructions. Every assay was carried out in triplicate. Cell proliferation rate (%) = (EdU-positive cells)/(total number of cells) × 100.

### Colony formation assay

Cells were plated on 35 mm dishes at a density of 6 × 10^2^ cells/plate. After cultivation for 14 days, the colonies were washed with phosphate-buffered saline (PBS) buffer and fixed with methanol for 20 min and stained with 0.1% crystal violet for 30 min. Colonies with more than 50 cells were counted.

### Soft agar assay

Soft agar assay was performed as previously described. Colonies were scored using an Olympus CKX41 microscope and data were analyzed using GraphPad Software.

### Wound healing assay

A scratch wound-healing assay was performed in 6-well plates. Scratch wounds were created using a sterile 200-μL pipette tip. The scratched area was marked and photographed at 0 h, 12 h and 24 h. The wound areas were calculated by ImageJ software.

### Transwell assay

Transwell invasion assay and migration assay were performed with a Transwell chamber coated with or without Matrigel in 24-well plates (Corning, MA, USA). The cells were seeded into the upper compartment of a 24-well chamber with an 8.0 μm pore (Corning, USA) with serum-free medium while the lower chambers were supplemented with medium containing 20% FBS as a chemoattractant. The cells were incubated for 48 h for the invasion assay or 24 h for the migration assay. Then, the cells migrated to the lower surface of the insert dish were fixed with 100% methanol, stained with 0.1% crystal violet and imaged under a microscope. The numbers of cells counted in five random fields were averaged.

### Cell cycle

Cells were cultured in a 6-well plate for 96 h. After trypsinization, the samples were washed with PBS buffer, fixed in 75% methanol, washed with staining buffer and resuspended in the staining buffer with 50 μg/ml RNase A and 50 μg/ml propidium iodide for 15 min at 4℃. DNA content of the cells was detected by flow cytometer. Data were analyzed by FCS software. All experiments were repeated three times.

### TdT-mediated dUTP-biotin nick end labelling (TUNEL) assay

TUNEL staining was assessed using a TUNEL Apoptosis Assay Kit (Beyotime Biotechnology, Haimen, China) following the manufacturer's instructions. TUNEL-positive cells were imaged with a fluorescent microscope. The cells with green fluorescence were considered as apoptotic cells. The percentage of cells positive for TUNEL was calculated using ImageJ software.

### Western blot

Thyroid cancer cells were harvested and lysed with RIPA buffer supplemented with a protease inhibitor (Beyotime Biotechnology, Haimen, China) to obtain protein according to the manufacturer's instruction. For Western blot assay, equal amount of protein was separated by SDS-PAGE, and transferred to a PVDF membrane (Millipore, Bedford, MA, USA). The membrane was blocked with 5% nonfat milk in Tris buffer saline/Tween 0.05% (TBST) for 2 h and incubated overnight at 4°C with a primary antibody of anti-PCNP antibody (1:1000, Abcam), anti-c-Jun N-terminal kinase (JNK) (1:1000, Cell signaling Technology), anti-extracellular signal-regulated protein kinase 1/2 (ERK1/2) (1:1000, Santa Cruz Biotechnology), anti-p38 (1:1000, Proteintech Group), anti-B-cell lymphoma-2 (Bcl-2) (1:1000, Proteintech Group), anti-B-cell lymphoma-extra-large (Bcl-xl) (1:1000, Proteintech Group), anti-Bcl-2-associated X protein (Bax) (1:1000, Proteintech Group), anti-Bcl-xl/Bcl-2-associated death promoter (Bad) (1:1000, Proteintech Group), anti-cleaved caspase-3 (1:1000, Proteintech Group), anti-cleaved caspase-8 (1:1000, Proteintech Group), anti-cleaved caspase-9 (1:1000, Proteintech Group), anti-cleaved PARP (1:1000, Proteintech Group), anti-LC3 (1:1000, Cell signaling Technology), anti-p62 (1:1000, Cell signaling Technology), anti-beclin-1 (1:1000, Cell signaling Technology), anti-Wnt3a (1:1000, Cell signaling Technology), anti-β-catenin (1:1000, Cell signaling Technology), anti-p-β-catenin (1:1000, Cell signaling Technology), anti-glycogen synthase kinase-3 beta (GSK-3β) (1:1000, Cell signaling Technology), anti-p-GSK-3β (1:1000, Cell signaling Technology) and anti-GAPDH (1:5000, Cell signaling Technology). After washing with TBST triply, the membrane was incubated with horseradish peroxidase (HRP)-conjugated secondary antibodies for visualization. The band images were analyzed with the Bio-Rad ChemiDoc XRS system.

### Xenograft nude mouse model

Thirty (n = 6 per group) 4 weeks old female BALB/c nude mice were purchased from the Beijing Vital River Laboratory Animal Technology Co., Ltd. (Certificate No. SCXK (Jing) 2011-0011, Beijing, China). For the tumor formation experiment, TPC-1 and ARO cells (4 × 10^6^ cells in 200 μl PBS) with stable over-expression or knockdown of PCNP were subcutaneously into the right flanks of 4 weeks old nude mice. Tumor size was monitored by measuring the length and width of the tumor every 4 days, and calculated with the formula: volume = 1/2 × length × width^2^. The mice were euthanized after 28 days, and tumors were weighed. Animal studies were approved by the Committee of Medical Ethics and Welfare for Experimental Animals of Henan University School of Medicine (HUSOM2020-276).

### Hematoxylin and eosin (HE) and IHC staining

The paraffin-embedded tissue sections were used for examination of HE staining. Briefly, tumor sections were fixed in 4% paraformaldehyde overnight and washed with PBS, then transferred to 70% ethanol, embedded in paraffin, cut and stained with HE. Then tumor tissues were photographed. IHC staining was performed using 4 μm paraffin-embedded tumor tissue sections. The primary antibodies included anti-Ki67, anti-CD31, anti-p21, anti-cleaved caspase-3, anti-beclin-1 were diluted to appropriate concentrations and then incubated overnight at 4℃. After careful washing, the slides were incubated with HRP conjugates using DAB detection.

### Statistical analysis

Data were presented as means ± standard error of the mean (SEM) of at least three independent experiments. Data were analyzed with one-way ANOVA analysis followed by Tukey's test using SPSS 17.0 software. *P* < 0.05 was considered as significant difference between indicated samples.

## Results

### PCNP is upregulated in thyroid cancer cells and related with clinicopathological features

**Figure 1:**
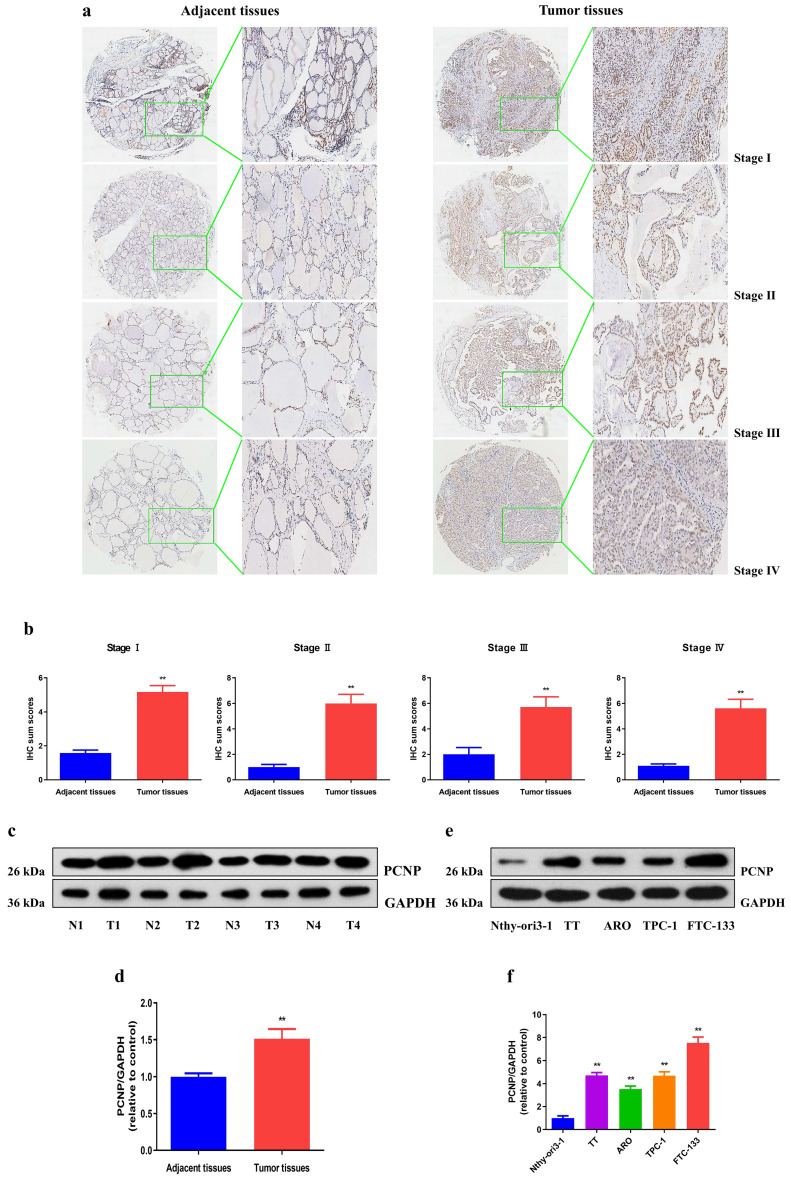
PCNP is overexpressed in human thyroid cancer tissues and cell lines. a, b Representative images (a) and quantification (b) of the expression of PCNP proteins assessed by IHC in different clinical stages of human thyroid cancer tissues and adjacent tissues (left: 400×; right: enlarged). c, d Western blot assay of PCNP protein and its relative expression level in fresh human thyroid cancer tissues (T) and adjacent normal tissues (N). e, f Western blot assay of PCNP protein and its relative expression level in TC cell lines and control Nthy-ori3-1. GAPDH was used as the loading control. Data are presented as mean ± SEM of three independent experiments; **P < 0.01 compared with adjacent normal tissues or Nthy-ori3-1 cells.

The overexpression of PCNP was confirmed by immunohistochemical analysis of microarray including 91 TC specimens and adjacent non-tumor patient samples (Fig. 1a, b). We further determined the protein level of PCNP in fresh surgical specimens of TC (Fig. 1c, d). In addition, to assess the clinical significance of PCNP, we evaluated the correlation of its expression with clinicopathological characteristics, as shown in Table 1, the PCNP level was associated with TNM stage (*P* = 0.03), tumor size (*P* = 0.039), and age (*P* = 0.003). However, there was no significant correlation between PCNP expression and other clinicopathological features, such as gender, metastasis and lymph nodes (*P* > 0.05). Furthermore, PCNP expression was detected in thyroid cancer cells (TT, ARO, TPC-1, FTC-133) and the normal human thyroid cell line Nthy-ori3-1. Significantly enhanced PCNP expression was observed in the thyroid cancer cells compared with that of the normal thyroid epithelium (Fig. 1e, f). Overall, these data imply that the high PCNP level may serve as an important regulator in TC development and progression.

### PCNP mediates the proliferation and viability of human TC cells

**Figure 2:**
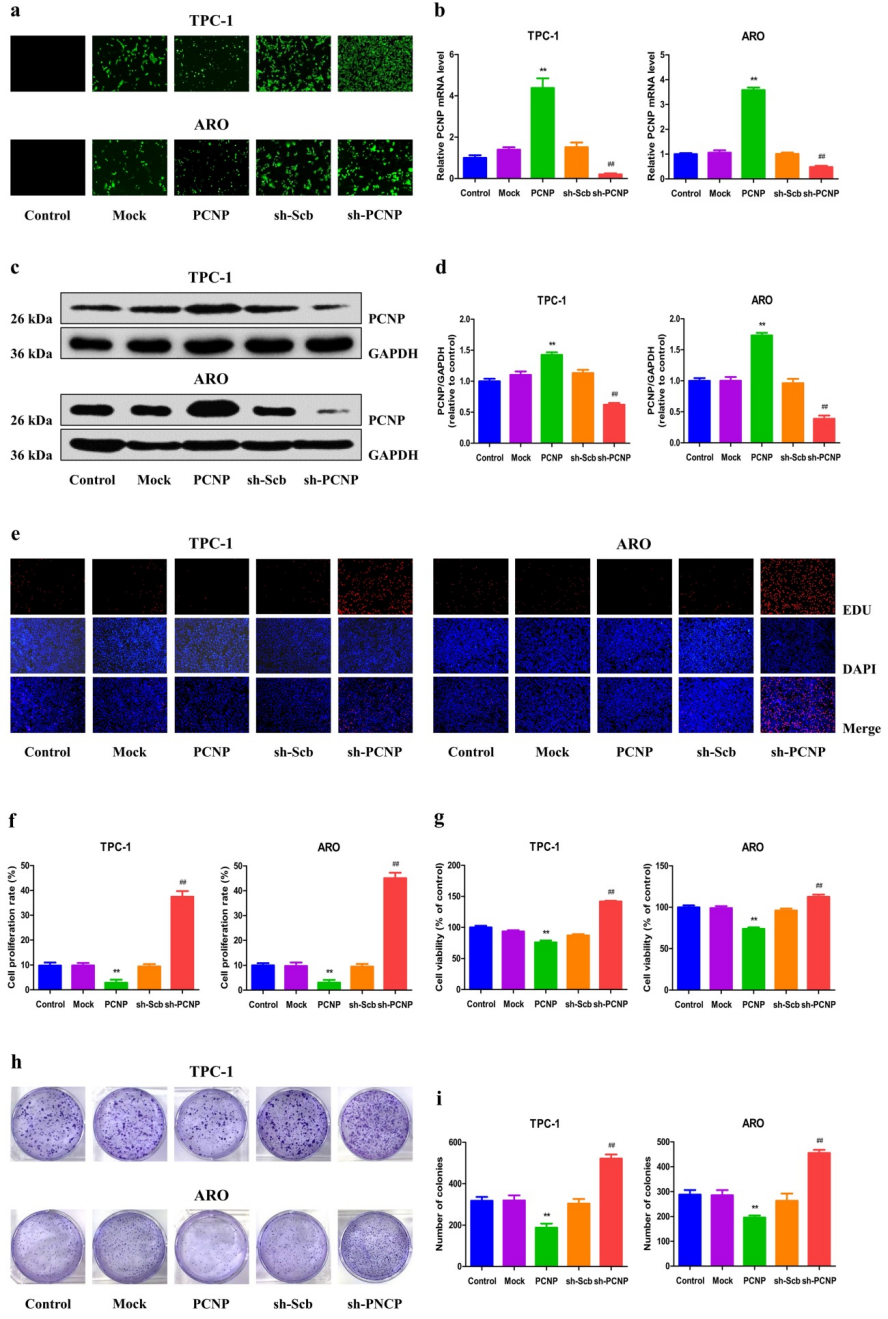
Effects of PCNP on the proliferation and viability of human thyroid cancer cells. a Fluorescence microscopy of PCNP in TPC-1 and ARO cells; original magnification 100×. b The expression level of PCNP mRNA was examined by RT-PCR. c The protein expression of PCNP was examined by Western blotting. GAPDH was used as the loading control. d The densitometry analysis of PCNP was performed, normalized to the corresponding GAPDH level. e DNA replication activities of TPC-1 and ARO cells in each group were examined by EdU assay; original magnification 100 ×. f The proliferation rate of each group was analysed. g The percentages of viable cells were determined using MTS assay and the cell viability of the control group was taken as 100%. h The clonogenic capacity was determined in TPC-1 and ARO cells. i The numbers of colonies were calculated. Data are presented as mean ± SEM of three independent experiments; **P < 0.01 compared with the Mock group; ^##^*P* < 0.01 compared with the sh‐Scb group.

To detect the role of PCNP in the growth of human TC cells, over-expression and knock-down experiments were performed. Transfection of PCNP into cells enhanced the PCNP level while transfection with a short hairpin RNA (shRNA) that targets PCNP (shPCNP) significantly reduced PCNP expression in TPC-1 and ARO cell lines (Fig. 2a, b). Consistently, similar trends were observed in the mRNA and protein levels of PCNP (Fig. 2c, d). As shown in Fig. 2e-g, after transfected with over-expressed PCNP, the drastically decreased cell proliferation and viability ability of TPC-1 and ARO was observed based on the EdU assay and MTS assay, while proliferation and viability were increased in TC cells transfected with down-expressed PCNP compared with control cells. Similarly, colony formation assay results revealed that clonogenic survival was reduction following transfection with upregulated PCNP while downregulated PCNP showed reverse trends (Fig. 2h, i).

### PCNP mediates the migration and invasion of human TC cells

**Figure 3:**
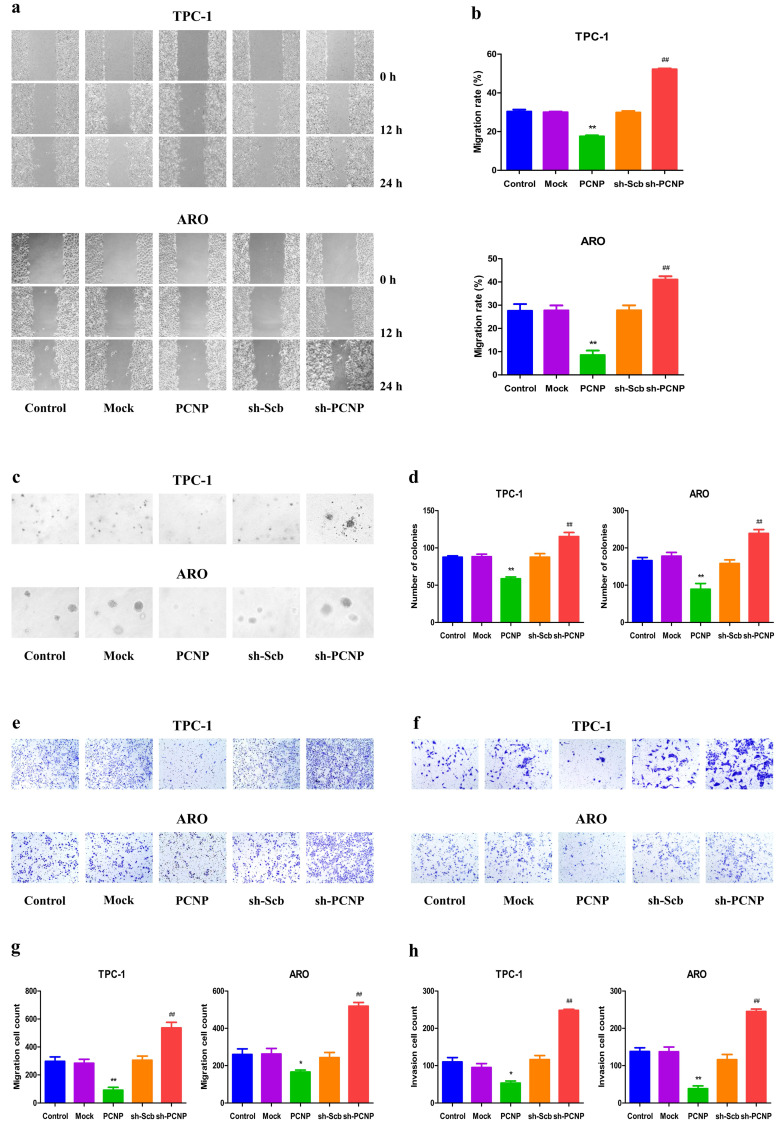
Effects of PCNP on the migration and invasion of human thyroid cancer cells. a The effect of PCNP on cell migration was measured by wound healing assay; original magnification 100 ×. b The migration rates of TPC-1 and ARO cells were calculated by the formula shown above. c Soft agar assay was performed to examine the anchorage-independent survival of cells; original magnification 100 ×. d The number of colonies was calculated. e Transwell assay was performed to assess the migration of TPC-1 and ARO cells; original magnification 200 ×. f Transwell assay was performed to assess the invasion of TPC-1 and ARO cells; original magnification 200×. g The numbers of the migrated cells were calculated. h The numbers of the invasive cells were calculated. Data are presented as mean ± SEM of three independent experiments; *P < 0.05, **P < 0.01 compared with the Mock group; ^##^*P* < 0.01 compared with the sh-Scb group.

Next, we transfected the PCNP overexpression vector or shPCNP into the TC cells to determine whether PCNP expression could influence cell migration and invasion. The wound healing assay showed that PCNP overexpression inhibited the migration of TPC-1 and ARO cells and PCNP knockdown exhibited the opposite effect (Fig. 3a, b). Furthermore, PCNP overexpression suppressed the anchorage- independent growth of TPC-1 and ARO cells, while a reverse trend was observed in the sh-PCNP group (Fig. 3c, d). In addition, the transwell assay revealed the migration and invasion capacities of TPC-1 and ARO cells were markedly improved in the sh-PCNP group, whereas the PCNP group showed opposite effects (Fig. 3e-h). These findings support the conclusion that PCNP exerts an important effect on the suppression of TC cell migration and invasion.

### Expression of PCNP leads to growth arrest

**Figure 4:**
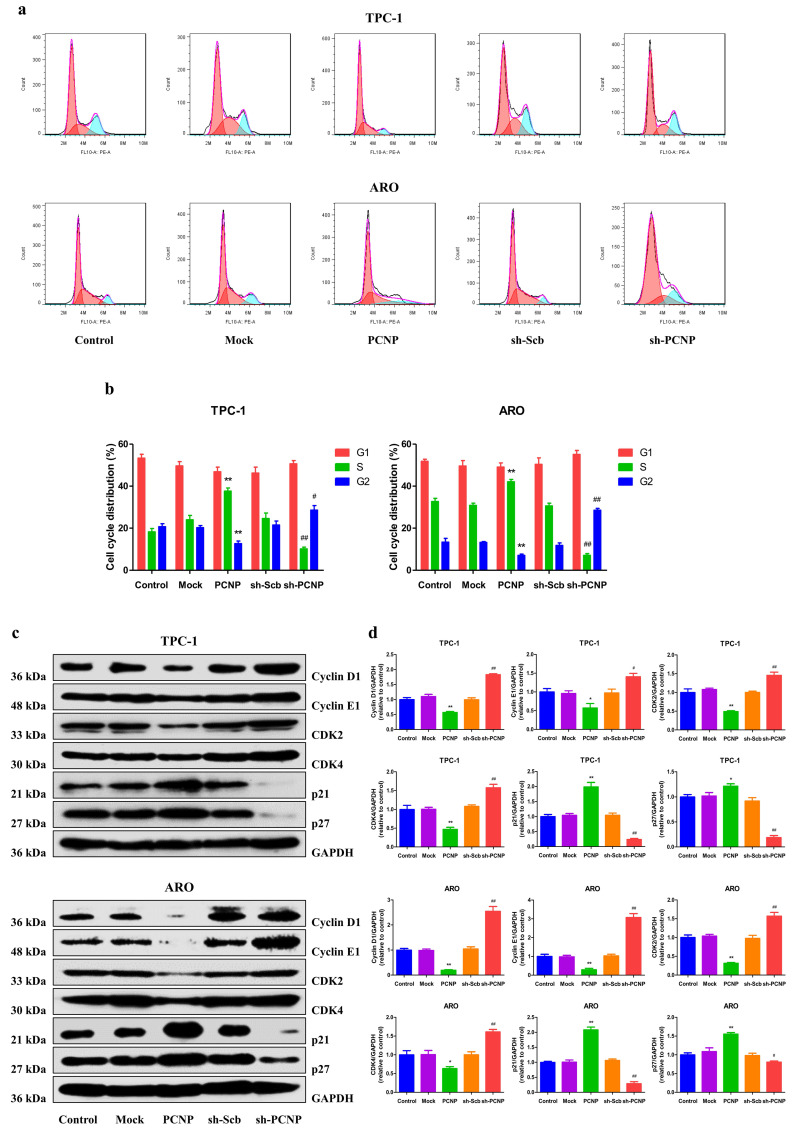
Effects of PCNP on the cell cycle of human thyroid cancer cells. a Cell cycle arrest effect was measured by flow cytometry. b Quantification of cell cycle data. c Western blotting analysis for the expression of Cyclin D1, Cyclin E1, CDK2, CDK4, p21 and p27 in TPC-1 and ARO cells. GAPDH was used as the loading control d The relative intensity of Cyclin D1, Cyclin E1, CDK2, CDK4, p21 and p27 by densitometry scanning are shown, normalized to the corresponding GAPDH level. Data are presented as mean ± SEM of three independent experiments; *P < 0.05, **P < 0.01 compared with the Mock group; ^#^*P* < 0.05, ^##^*P* < 0.01 compared with the sh-Scb group.

To further explore whether the cell cycle progression alteration contributes to the effect of PCNP overexpression or knockdown on cell viability, flow cytometric analysis was used to examine cell cycle progression. PCNP upregulation displayed an elevated cell cycle arrest at the S phase and PCNP downregulated showed a cell cycle arrest at the G2 phase (Fig. 4a, b). Moreover, protein levels of the cell cycle related genes p21 and p27 were significantly increased while Cyclin D1, Cyclin E1, CDK2 and CDK4 were significantly decreased in the PCNP group compared with the Mock group. In contrast, PCNP knockdown cells showed conversed results. These results confirm that the PCNP mediates TC cells proliferation attributed to cell cycle arrest at the S checkpoint.

### PCNP promotes apoptosis through activating the mitogen-activated protein kinase (MAPK) pathway in human TC cells

**Figure 5:**
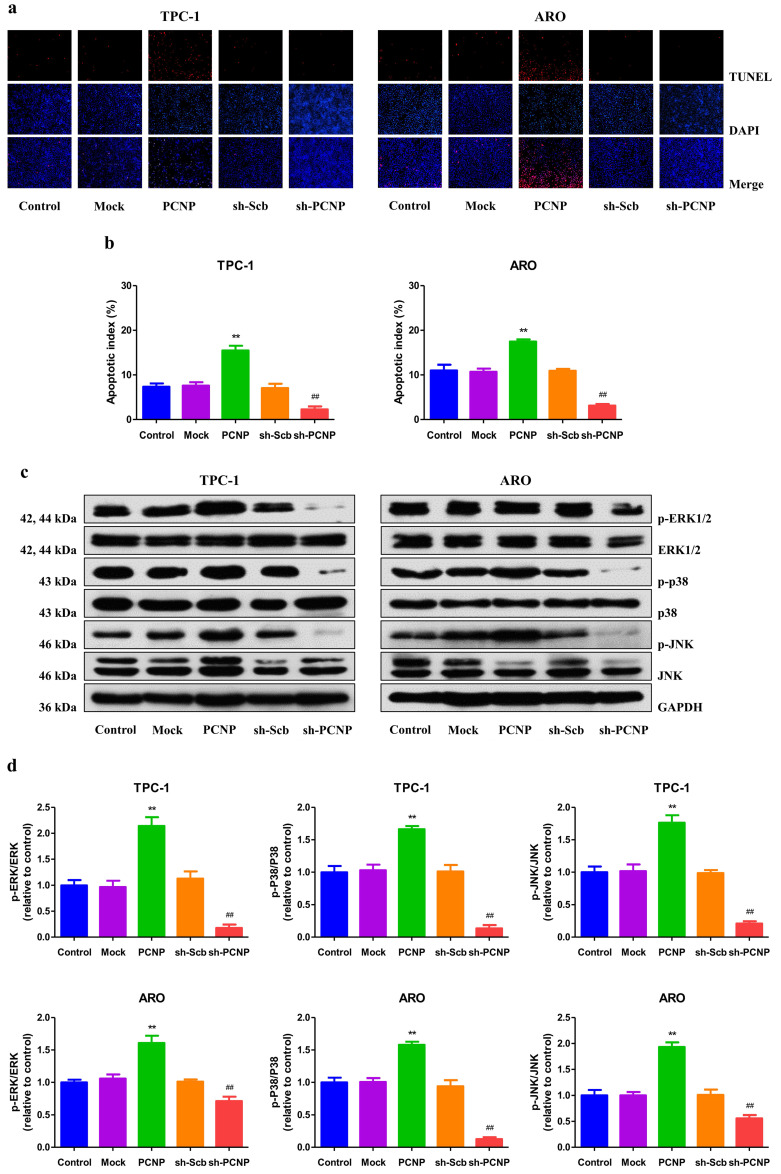
Effects of PCNP on the apoptosis and MAPK signaling pathway in human thyroid cancer cells. a The apoptotic levels of TPC-1 and ARO cells were measured by TdT-mediated dUTP-biotin nick end labeling (TUNEL) staining; original magnification100 ×. b The percentages of TUNEL-positive cells were calculated. c Western blotting analysis for the expression of p-ERK1/2, ERK1/2, p-JNK, JNK, p-P38 and P38 in TPC-1 and ARO cells. GAPDH was used as the loading control. d The densitometry analyses of p-ERK1/2, ERK1/2, p-JNK, JNK, p-P38 and P38 were performed in TPC-1 and ARO cells, normalized to the corresponding GAPDH level. Data are presented as mean ± SEM of three independent experiments; **P < 0.01 compared with the Mock group;^ ##^*P* < 0.01 compared with the sh-Scb group.

To explore the potential role of PCNP in regulation of apoptosis, we performed the TUNEL assay on TPC-1 and ARO cells. The results demonstrated that in PCNP group, the apoptotic index was significantly higher than that in mock group, while lower in the sh-PCNP group than sh-scb group (Fig. 5a, b). In addition, the expression of pro-apoptotic proteins of cleaved caspase-3, cleaved caspase-8, cleaved caspase-9, cleaved PARP, Bax, and Bad in human TC cells exhibited similar elevation trends and the reverse expression levels of anti-apoptotic protein Bcl-2 and Bcl-xl (Fig. S1). MAPKs, which include the ERK, c-Jun N-terminal kinases (JNK) and p38, have been reported to be associated with cellular apoptosis^12^. In the study, western blot analysis revealed that, all the three genes (ERK1/2, p38, JNK) were activated by PCNP; yet PCNP knock-down had an opposite effect (Fig. 5c, d). Together, PCNP may possess the pro-apoptotic capacity via MAPK pathway in human TC cells.

### PCNP modulates autophagy via the Wnt/β-catenin pathway in human TC cells

**Figure 6:**
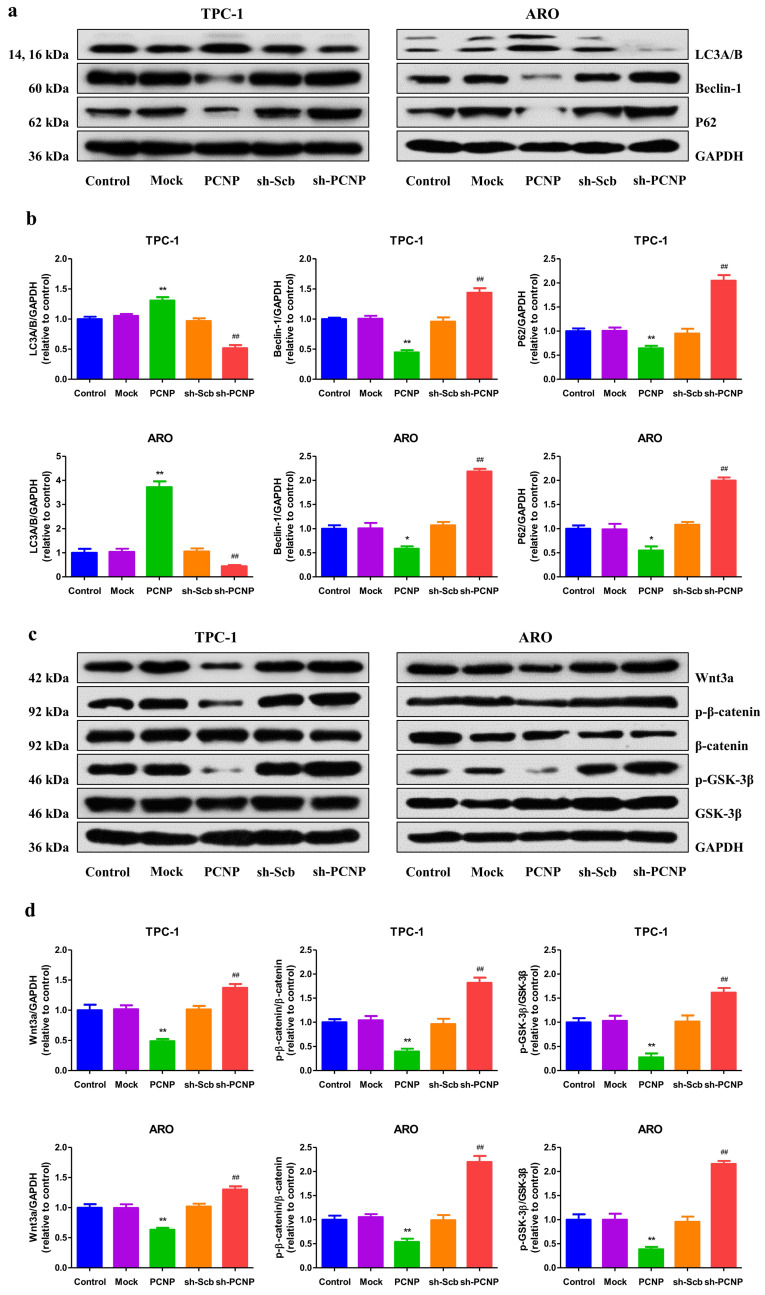
Effects of PCNP on the autophagy and Wnt/β-cantenin signaling pathway in human thyroid cancer cells. a Western blotting analysis for the expression of LC3A/B and P62 in TPC-1 and ARO cells. GAPDH was used as the loading control. b The densitometry analyses LC3A/B and P62 were performed in TPC-1 and ARO cells, normalized to the corresponding GAPDH level. c Western blotting analysis for the expression of Wnt3a, p-β-cantenin, β-cantenin, p-GSK-3β and GSK-3β in TPC-1 and ARO cells. GAPDH was used as the loading control. d The densitometry analyses of Wnt3a, p-β-cantenin, β-cantenin, p-GSK-3β and GSK-3β in TPC-1 and ARO cells, normalized to the corresponding GAPDH level. Data are presented as mean ± SEM of three independent experiments; *P < 0.05, **P < 0.01 compared with the Mock group; ^##^*P* < 0.01 compared with the sh-Scb group.

Autophagy and apoptosis often occur in the same cell and autophagy frequently precedes apoptosis^13^. Autophagy may have either a positive or a negative effect on tumor growth in a context‐dependent fashion^14^. Our results showed that PCNP upregulation showed decreased trends in the protein levels of autophagic markers p62 and beclin 1, and an increase in LC3A/B, while PCNP downregulated exhibited a reverse trend (Fig. 6a, b). Moreover, autophagy was recently implicated in the regulation of Wnt/β-catenin pathway. The western blot results indicated that PCNP overexpression restrained the up-regulation of Wnt3a and the activation of GSK-3β and β-catenin, whereas PCNP knockdown exerted opposite effects (Fig. 6c, d). These results together suggest that PCNP modulates autophagy via the Wnt/β-catenin pathway in human TC cells.

### PCNP regulates the growth of human TC xenograft tumors in nude mice

**Figure 7:**
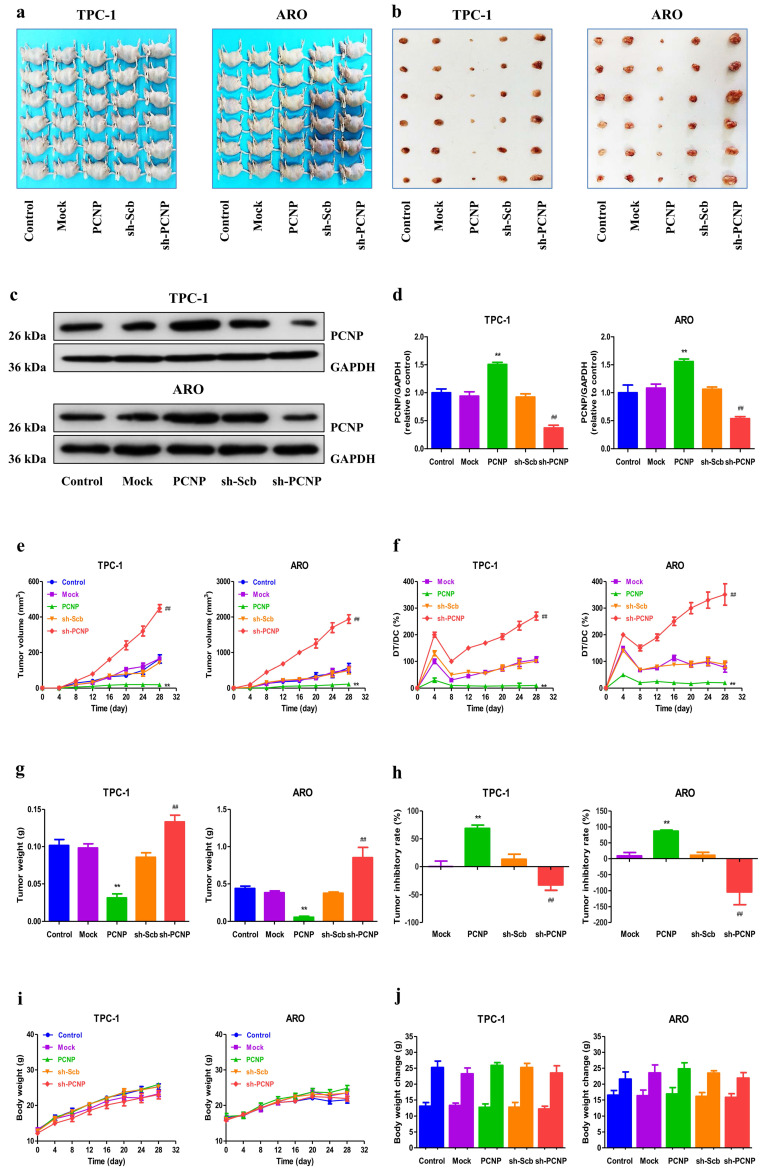
Effects of PCNP on the growth of TPC-1 and ARO xenograft tumors in nude mice. a,b Representative xenografts dissected from different groups of nude mice were shown. c Western blotting analysis for the expression of PCNP in tumors in nude mice of TPC-1 and ARO, GAPDH was used as the loading control. d The densitometry analyses of PCNP, normalized to the corresponding GAPDH level. e, f The tumor volume of each group was measured every day and the TVDT was calculated by the formula shown above. g, h The tumors were weighed and the inhibition rates of tumor growth were calculated by the formula shown above. i, j The bodyweight change curve of each group during the experiment and the bodyweight of each group on the first day (day 0) and the last day (day 28). Values are presented as mean ± SEM (n = 6); **P < 0.01 compared with the Mock group; ^##^*P* < 0.01 compared with the sh‐Scb group.

To investigate whether PCNP was involved in TC tumor progression *in vivo*, we established a mouse TC xenograft model by injecting PCNP-overexpression cells or PCNP-knockdown cells (shPCNP) into nude mice. As the representative images of tumor exhibited in Fig. 7a, b, the tumors derived from PCNP-overexpressing cells were smaller than the control tumors whereas the tumors derived from PCNP-abrogation cells were bigger than the control tumors. We also used western blot analysis to confirm the efficacy of PCNP overexpression or knockdown at protein level (Fig. 7c, d). Notably, PCNP overexpression significantly reduced the growth of xenograft tumors when compared with the Mock group. Nevertheless, PCNP knockdown promoted the growth of xenograft tumors compared to the sh-Scb group. Tumor growth curves, DT/DC%, and the measured tumor weights were presented in Fig. 7e-g. The inhibition rate analyses further showed the promotion of PCNP in tumor growth (Fig. 7h). Besides, no significant difference was detected in body weight among groups (Fig. 7i, j). In sum, these results show that PCNP could mediate the growth of human TC.

### PCNP is associated with proliferation, angiogenesis, cell-cycle arrest, apoptosis and autophagy

**Figure 8:**
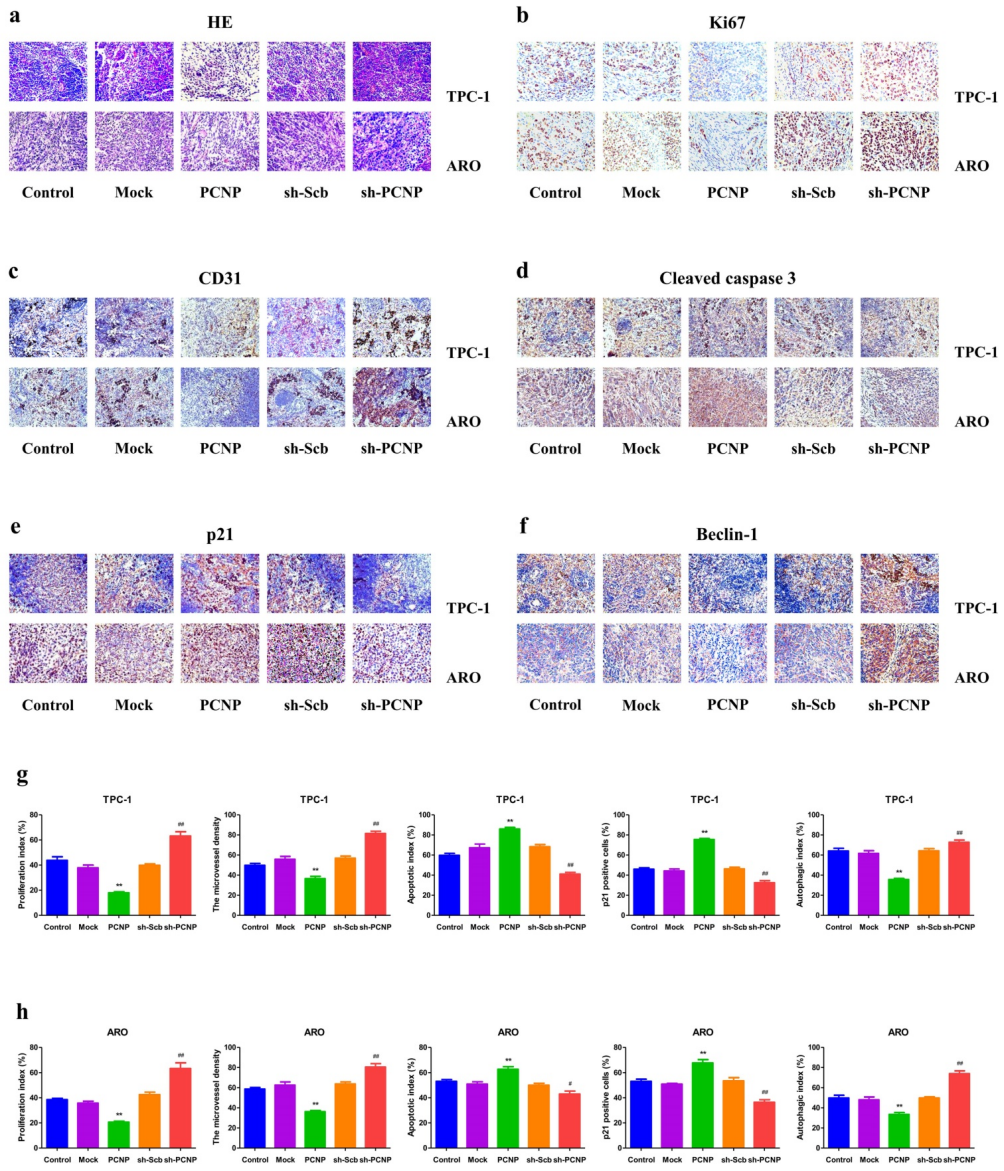
HE and Immunohistochemistry analysis of TPC-1 and ARO xenograft tumors. a HE staining of paraffin sections of nude mice in TPC-1 and ARO. b-f Immunohistochemistry examination of Ki67, CD31, cleaved-caspase3, p21, and beclin1 expression in thyroid cancer tissues of nude transplanted tumor. g The quantification of the expression of Ki67, CD31, cleaved-caspase-3, p21, beclin1 expression by IHC. Values are presented as mean ± SEM (n = 6); **P < 0.01 compared with the Mock group; ^#^*P* < 0.05, ^##^*P* < 0.01 compared with the sh-Scb group.

As shown in Fig. 8a, HE staining results revealed that PCNP was negatively correlated to tumor malignancy and metastasis. Inhibition of tumor growth was also shown by staining for a decrease in proliferating cell (Ki67 staining) and the microvessel density (CD31 staining) (Fig. 8b, c). Moreover, PCNP upregulation significantly stimulated apoptosis based on IHC of apoptotic (TUNEL) marker and a reverse effect was observed in the sh-PCNP group (Fig. 8d). Additionally, in IHC results, p21 expression levels were obviously declined in the PCNP knock-down group compared with the sh-control group; the opposite effects were shown in the PCNP overexpression group (Fig. 8e). The results of autophagic index confirmed that increased PCNP expression promoted the *in vivo* autophagy of TC cells and decreased PCNP expression produced conversed effect (Fig. 8f).

## Discussion

In previous years, increasing attention has been focused on PCNP. As a nuclear protein rich in PEST sequence, PCNP is strictly controlled by the ubiquitination- proteasome degradation pathway, and is also a rapidly degraded protein sequence with a short half-life^7^. Similar to other nuclear genes containing PEST motif (P53, PTEN), PCNP serves an important role in regulation of the process of tumor. For example, our previous study revealed that PCNP mediated the growth of human neuroblastoma cells via MAPK and PI3K/Akt/mTOR pathways^9^. Also, PCNP was reported to play a vital role in growth, migration and invasion in lung cancer^11^ and ovarian cancer^10^. PCNP can have oncogene or tumor suppressor qualities varying among types of malignancies depending on tissue-specific and cellular contexts. As a transcription factor, PCNP can play dual roles in different tumor types by controlling the expression of multiple genes, as well as their controlled signaling pathways. In addition, it is reported that p53 is the most extensively studied tumor suppressor gene, and mutant p53 proteins not only lose their tumor suppressive abilities, but also gain additional oncogenic functions that provide cells with growth and survival advantages^15^, ^16^. By this reasoning, the mutation of PCNP may also cause the controversial roles of PCNP in some other cancers.

However, the role of PCNP in TC is still unclear. In this study, our results suggested that PCNP level was higher in TC tissue compared with adjacent non-tumor tissue. Furthermore, PCNP expression was associated with T classification of TC. In light of the results, we can conclude that PCNP may be involved in the procession of TC. In the present study, ARO and TC-1 cells were used to detect the mechanism of PCNP on the growth of TC.

Functionally, our results demonstrated that PCNP over-expression attenuated the proliferation and viability, as well as inhibited the migration and invasion capabilities of TC-1 and ARO cells, whereas PCNP knockdown exhibited completely opposite effects, suggesting that PCNP could play important roles in the growth, migration, and invasion of human TC cells. Dysregulation of cell cycle regulators contributes to limitless tumor cell growth and proliferation^17^. Yang et al proposed that Heme Oxygenase-1 inhibitors suppressed tumor growth by inducing cell cycle arrest and in thyroid cancer cells^18^. Notably, it has been reported that PCNP may be involved in the signaling pathway concerned with cell cycle regulation and/or genome stability by interacting with NIRF^6^, ^19^. In our study, we found that PCNP induced S phase arrest of TC-1 and ARO cells, which might eventually decelerate TC growth. In contrast to this, a decrease in PCNP reduced the S population of cell cycle which might finally promote cell progression.

As a programmed cell death, apoptosis is a critical process to maintain cell and tissue homeostasis^20^. There are two apoptotic signaling pathways: extrinsic stimulation of death receptors and the intrinsic mitochondrial pathway^21^. The activation of caspase and Bax causes mitochondrial dysfunction and morphological changes, thus promoting mitochondrial apoptosis, which is blocked by Bcl-2, an anti-apoptotic protein^22^. Our results showed that PCNP over-expression remarkably increased the apoptotic index, protein expressions of Bax/Bcl-2 and Bad/Bcl-xl ratios and activated caspase cascade induced PARP, caspase-9 and caspase-3, but not caspase-8, suggesting the activation of mitochondria-mediated pathway. Of note, caspase-8 is not only the initiator caspase of extrinsic apoptosis but also the inhibitor of necroptosis mediated by RIPK3 and MLKL^23^. Additional inhibition of the proteolytic enzyme caspase-8 by microbes or pharmacological agents triggers the necroptotic pathway^24^. Our data showed caspase-8 even decreased in PCNP overexpressed group compared the control which may indicate necroptosis was also associated with PCNP-induced the cell death. Together, PCNP may promote cell death by the activation of mitochondria-mediated pathway and necroptotic pathway. Mitogen-activated protein kinases (MAPKs) play an important role in the process of intracellular signal transduction by managing cellular responses to a diverse array of stimuli that regulate cell functions, including cell proliferation, differentiation, mitosis and apoptosis^25^, ^26^. ERK, JNK and p38 MAPK are key members of the MAPK family that are associated with the induction of cell cycle arrest and apoptosis^27^, ^28^. The mechanism of PCNP-induced cell apoptosis was further investigated by assessing the role of ERK, JNK and p38 in TPC-1 and ARO cells. The results indicated that PCNP overexpression could activate ERK, JNK and p38 thereby promote apoptosis in TC revealing that PCNP mediated mitochondria-dependent apoptotic pathway in human thyroid cancer cells.

Autophagy, type II programmed cell death, is a dynamic degradation procedure involving cytoplasmatic vesicles and intracellular organelles are transported into vacuoles called autophagosomes^29^, ^30^. A lot of studies have demonstrated a very complex association between apoptosis and autophagy^31^, ^32^. Many findings have suggested an important role of autophagy in TC^14^, ^33^, ^34^. According to recent reports, Wnt/β-catenin pathway has been the most well depicted in various cancers, including thyroid carcinoma^35^-^37^. Moreover, Wnt/β-catenin signaling plays an important role during development, adult tissue homeostasis and tumorigenesis and is identified as a negative regulator of both basal and stress-induced autophagy in colorectal carcinoma^38^. Our results showed that PCNP overexpression promoted autophagy by inhibiting the activation of Wnt/β-catenin pathway proteins, indicating that PCNP could modulate autophagy through the Wnt/β-catenin signaling pathway in human TC cells.

In addition to our *in vitro* studies, we also examined the role of PCNP *in vivo*. Our data revealed that PCNP inhibits tumor growth in mice. In addition, injection was not associated with any significant toxicity, since no differences in body weight. The remarkable tumor suppressor capacity of PCNP* in vivo* was confirmed by IHC analysis for proliferation (Ki67), associated angiogenesis (CD31), apoptosis (cleaved caspase-3), cell cycle (p21), autophagy (Beclin-1) of tumor sections from tumors harvested from control, Mock, PCNP, sh-sub, and sh-PCNP group mice.

PCNP may act as a transcription factor. However, it remains unclear what exactly direct targets of PCNP are. PCNP with the presence of high-score PEST sequences and potential phosphorylation sites is reminiscent of key molecule governing cell-cycle progression and transcriptional regulation. Moreover, transcription patterning analysis of a PCNP showed that it is co-expressed with MARCH7, BMI1, TMEM123, TRAM1, and PSMC6^39^. Whereas, in searching for PCNP-associating direct targets, ChIP or ChIP-seq experiment should be conducted for validation. Collectively, our findings reveal PCNP can mediate the proliferation, migration, and invasion of human thyroid cancer cells through MAPK and Wnt/β-catenin signaling pathways which finally contribute to the development of TC. Given that the role of PCNP in the developmental process of human TC cells, it could be a potential therapeutic target for TC.

## Supplementary Material

Supplementary figure.

## Figures and Tables

**Table 1:** Association between PCNP expression and clinicopathological characteristics of patients with thyroid carcinoma (n = 91).

Characteristics	Cases	PCNP expression		*P* value
		Low	High	
**Age (years)**				0.003
≤44	42	20	22	
45-59	38	17	21	
≥60	11	7	4	
**Gender**				0.986
Male	36	18	18	
Female	55	24	31	
**Tumor size (cm)**				0.039
≤3	78	33	45	
>3	13	12	1	
**T classification**				0.03
T1	41	15	26	
T2	40	22	18	
T3	7	3	4	
T4	3	1	2	
**Lymph node status**				0.389
N0	47	21	26	
N1	44	20	24	
**Metastasis**				0.388
M0	90	43	47	
Mx	1	0	1	
