# Supplementary material for: PCNP is a novel regulator of proliferation, migration, and invasion in human thyroid cancer
Source: Int J Biol Sci. 2022 May 16;18(9):3605–20. doi: 10.7150/ijbs.70394 (PMC9254465; doi:10.7150/ijbs.70394)
Supplement: Supplementary file 1 — Supplementary figure. [file ijbsv18p3605s1.pdf]

Figure S1. Effects of PCNP on the expression of pro- and anti-apoptotic proteins. a Western blotting analysis for the expression of cleaved caspase-3, cleaved caspase-8, cleaved caspase-9, cleaved PARP, Bax, Bcl-2, Bad and Bcl-xl in TPC-1 and ARO cells. GAPDH was used as the loading control. b, c The densitometry analyses of cleaved caspase-3, cleaved caspase-8, cleaved caspase-9, cleaved PARP, Bax, Bcl-2, Bad and Bcl-xl in TPC-1 and ARO cells, normalized to the corresponding GAPDH level. Values are presented as mean ± SEM of three independent experiments; $^{*}P < 0.05$, $^{**}P < 0.01$ compared with the Mock group; $^{\#}P < 0.05$, $^{\#\#}P < 0.01$ compared with the sh-Scb group.

Figure S1

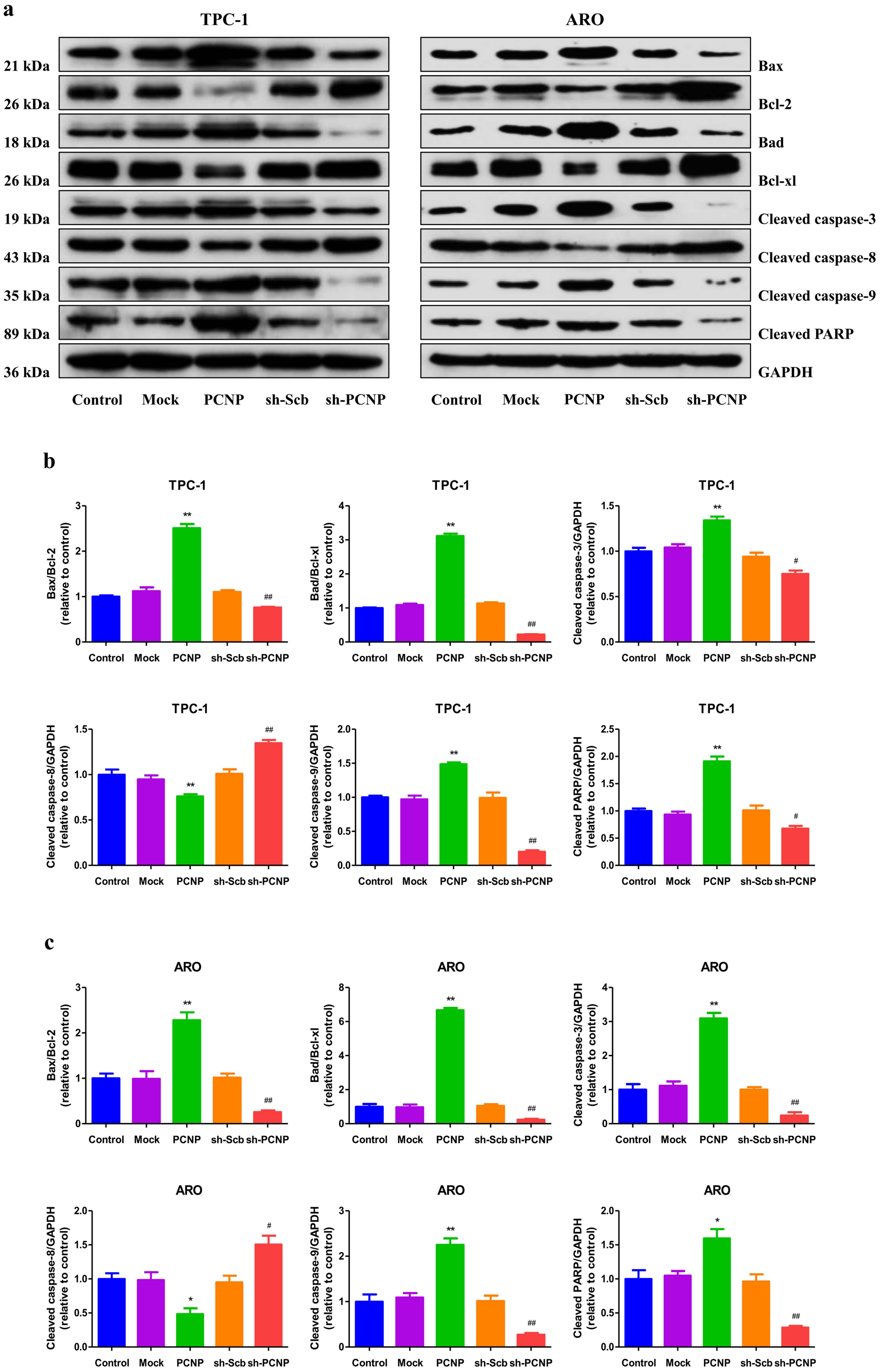
a
TPC-1
ARO
21 kDa
26 kDa
18 kDa
26 kDa
19 kDa
43 kDa
35 kDa
89 kDa
36 kDa
Bax
Bcl-2
Bad
Bcl-xl
Cleaved caspase-3
Cleaved caspase-8
Cleaved caspase-9
Cleaved PARP
GAPDH
Control Mock PCNP sh-Scb sh-PCNP
Control Mock PCNP sh-Scb sh-PCNP
b
TPC-1
Bax/Bcl-2 (relative to control)
Bad/Bcl-xl (relative to control)
Cleaved caspase-3/GAPDH (relative to control)
Cleaved caspase-8/GAPDH (relative to control)
Cleaved caspase-9/GAPDH (relative to control)
Cleaved PARP/GAPDH (relative to control)
Control Mock PCNP sh-Scb sh-PCNP
**
##
#
c
ARO
Bax/Bcl-2 (relative to control)
Bad/Bcl-xl (relative to control)
Cleaved caspase-3/GAPDH (relative to control)
Cleaved caspase-8/GAPDH (relative to control)
Cleaved caspase-9/GAPDH (relative to control)
Cleaved PARP/GAPDH (relative to control)
Control Mock PCNP sh-Scb sh-PCNP
*
**
#
##
